# Durable Expansion of TCR-δ Meta-Clonotypes After BCG Revaccination in Humans

**DOI:** 10.3389/fimmu.2022.834757

**Published:** 2022-03-30

**Authors:** Charlotte A. James, Krystle K. Q. Yu, Koshlan Mayer-Blackwell, Andrew Fiore-Gartland, Malisa T. Smith, Erik D. Layton, John L. Johnson, Willem A. Hanekom, Thomas J. Scriba, Chetan Seshadri

**Affiliations:** ^1^ Department of Medicine, University of Washington, Seattle, WA, United States; ^2^ Vaccine and Infectious Disease Division, Fred Hutchinson Cancer Research Center, Seattle, WA, United States; ^3^ Tuberculosis Research Unit, Department of Medicine, Case Western Reserve University and University Hospitals Cleveland Medical Center, Cleveland, OH, United States; ^4^ South African Tuberculosis Vaccine Initiative and Institute of Infectious Disease and Molecular Medicine, Division of Immunology, Department of Pathology, University of Cape Town, Cape Town, South Africa; ^5^ Tuberculosis Research and Training Center, University of Washington, Seattle, WA, United States

**Keywords:** BCG - Bacille Calmette-Guérin vaccine, tuberculosis, gamma delta (γδ) T cells, donor unrestricted T cells, MAIT cell, iNKT cell

## Abstract

*Mycobacterium bovis* bacille Calmette-Guérin (BCG) has been used for 100 years and prevents disseminated tuberculosis and death in young children. However, it shows only partial efficacy against pulmonary tuberculosis (TB) in adults, so new vaccines are urgently needed. The protective efficacy of BCG depends on T cells, which are typically activated by pathogen-derived protein antigens that bind to highly polymorphic major histocompatibility complex (MHC) molecules. Some T cells recognize non-protein antigens *via* antigen presenting systems that are independent of genetic background, leading to their designation as donor-unrestricted T (DURT) cells. Whether live whole cell vaccines, like BCG, can induce durable expansions of DURT cells in humans is not known. We used combinatorial tetramer staining, multi-parameter flow cytometry, and immunosequencing to comprehensively characterize the effect of BCG on activation and expansion of DURT cell subsets. We examined peripheral blood mononuclear cells (PBMC) derived from a Phase I study of South African adults in which samples were archived at baseline, 3 weeks, and 52 weeks post-BCG revaccination. We did not observe a change in the frequency of total mucosal-associated invariant T (MAIT) cells, invariant natural killer T (iNKT) cells, germline encoded mycolyl-reactive (GEM) T cells, or γδ T cells at 52 weeks post-BCG. However, immunosequencing revealed a set of TCR-δ clonotypes that were expanded at 52 weeks post-BCG revaccination. These expanded clones expressed the Vδ2 gene segment and could be further defined on the basis of biochemical similarity into several ‘meta-clonotypes’ that likely recognize similar epitopes. Our data reveal that BCG vaccination leads to durable expansion of DURT cell clonotypes despite a limited effect on total circulating frequencies in the blood and have implications for defining the immunogenicity of candidate whole cell TB vaccines.

## Introduction

*Mycobacterium tuberculosis* (M.tb), the causative agent of tuberculosis (TB), was responsible for 10 million new cases and 1.4 million deaths worldwide in 2019 (1). *M. bovis* bacille Calmette-Guérin (BCG) is the only licensed vaccine for TB, and provides protection against disseminated forms of the disease in children, but shows variable efficacy in preventing pulmonary TB in adults (2, 3). As TB is transmitted through airborne droplets, adolescents and adults with pulmonary TB are thought to be the primary transmitters of the disease, rendering the current BCG vaccination strategy unable to control the epidemic. The development of new vaccines that have demonstrated efficacy against pulmonary TB is thus a priority for the field.

Studies in murine and non-human primate (NHP) models have demonstrated an essential role for T cell responses in protection against M.tb challenge (4). However, it is not known which pathogen-derived antigens are targeted by protective T cells. Canonically, T cells are activated by foreign peptide antigens when bound to highly polymorphic major histocompatibility complex (MHC) molecules. Peptide subunit vaccines are highly immunogenic, but there has been mixed efficacy in prevention of M.tb infection and TB disease (5, 6). More recently, T cells have been shown to recognize non-peptide antigens through MHC-independent modes of recognition. For example, T cells are activated by lipids and small molecules presented by cluster of differentiation 1 (CD1) and major histocompatibility complex (MHC)-related protein 1 (MR1), respectively (7, 8). Further, T cells with a γδ T cell receptor (TCR) can recognize non-peptide antigens presented by butyrophilin molecules, CD1, and MR1 (9–12). Vγ9Vδ2 T cells are the most abundant subset of γδ T cells in human blood and are activated by phosphoantigens present in tumors and bacteria (13, 14). Because CD1, MR1, and butyrophilin exhibit limited sequence diversity, the T cells that act through these systems are called donor-unrestricted T (DURT) cells and express semi-invariant TCRs (15). The two highest frequency semi-invariant DURT cell populations in human blood are invariant natural killer T (iNKT) cells that recognize lipids presented by the CD1d isoform and mucosal associated invariant T (MAIT) cells that recognize metabolite antigens presented by MR1 (7, 16–21).

DURT cell populations have been associated with the immune response to BCG and M.tb in murine and non-human primate studies. In a murine model of pulmonary BCG infection, MAIT cells were recruited to the lung, which was associated with reduced bacterial burden (22). Induced MAIT cells can also inhibit BCG replication in the lungs of infected mice (23). Further, NKT cells are activated and proliferate following BCG infection in mice, but NKT cell frequency returns to baseline following infection (24). In non-human primates, BCG vaccination induces a robust expansion of γδ T cells and mycobacterial lipid-specific T cells and induces activation of MAIT cells (25–27). In fact, adoptive transfer of γδ T-cells in a non-human primate model of M.tb-infection reduced the bacterial burden and prevented dissemination of disease (28). Finally, in a study of BCG-vaccinated rhesus macaques, CD8+ iNKT cells were expanded in animals that were able to control subsequent M.tb infection (29). Taken together, these data highlight a potential role for DURT cells in mediating a protective immune response to BCG vaccination in animal models. Whether whole cell vaccines, such as BCG, modulate DURT cells in humans is unknown.

Here, we sought to comprehensively evaluate changes in DURT cell populations after BCG vaccination in humans. We leveraged samples collected in the context of a Phase I study of the safety and reactogenicity of BCG revaccination, with or without INH pre-treatment, in South African adults with latent TB (30). Peripheral blood mononuclear cells (PBMC) were archived at baseline or 3 weeks and 52 weeks post-BCG. We employed combinatorial tetramer staining and multi-parameter flow cytometry to define the frequencies and phenotypes of DURT cells. We also used immunosequencing of TCR-α and TCR-δ chains to define specific TCR rearrangements, or clonotypes, that undergo expansion after BCG vaccination. Surprisingly, we found no durable changes in DURT cell frequencies using flow cytometry. However, we were able to define TCR-δ clonotypes that were expanded in many individuals up to one year after BCG vaccination. Our data thus reveal that BCG induces sustained changes in the DURT cell repertoire that may contribute to a protective immune response.

## Materials and Methods

### Clinical Cohorts

Healthy 18 to 40-year-old South African adult participants were recruited from Worcester in the Western Cape, South Africa. All participants were strongly TST positive (≥ 15mm induration when tested with PPD RT-23), human immunodeficiency virus (HIV)-seronegative, and received BCG at birth. All participants had a baseline BCG scar and large TST induration (>15mm), thus mitigating against poor baseline responses to BCG. Additionally, participants were screened for the presence of active tuberculosis using chest radiography, sputum microscopy and culture (30). These individuals participated in a phase I clinical trial to evaluate the safety and reactogenicity of BCG revaccination, with or without INH pre-treatment, and were randomized in parallel into two groups as previously described (30). Participants included in the present study received a course of 6 months of isoniazid within a maximum period of 7 months, followed by BCG vaccination and a subsequent period of observation. Danish strain 1331 BCG Vaccine SSI (Statens Serum Institut, Copenhagen, Denmark) was administered intradermally at an adult dose of 2-8×10^5^ CFU. Isoniazid (Westward Pharmaceutical Corporation, Eatontown, NJ, USA) was administered daily at 5 mg/kg rounded up to the nearest 100 mg, with a maximum dose of 300 mg per day. Isoniazid adherence was monitored by pill counts at clinic visits and random urine isoniazid metabolite testing. Heparinized whole blood was collected from participants and processed within 45 minutes of phlebotomy, as previously described (31), at enrolment, 1 month after isoniazid preventive therapy initiation, at BCG vaccination, at 3 and 5 weeks, and 52 weeks post-vaccination. For this study, PBMC donated at enrolment, BCG vaccination, 3 weeks post-vaccination, and 52 weeks post-vaccination were analyzed. The 3 week post-vaccination time point was selected because previous data demonstrate that classical BCG-induced CD4+ T cell responses are detectable at this time point (32, 33). Samples were chosen from the parent study on the basis of availability of cryopreserved PBMC at each of the three time points tested. The distribution of age, sex, and body mass index (BMI) was similar between the parent study and samples tested in this study (**Supplemental Table 1**) (30).

### Generation of GMM-CD1b, MA-CD1b, α-GalCer-CD1d, and 5-OP-RU-MR1 Tetramers

Soluble biotinylated CD1b monomers were provided by the National Institutes of Health Tetramer Core Facility (Emory University, Atlanta, GA). The loading protocol for CD1b monomers was based on previously published loading protocols using a 40-fold molar excess of mycolic acid (MA) and 100-fold molar excess of glucose monomycolate (GMM) (34–36). For GMM-loaded CD1b tetramers, C32-GMM derived from *Rhodococcus equi* was dried down and sonicated into 50 mM sodium citrate buffer at pH 4, containing 0.25% CHAPS. For MA-loaded tetramers, synthetic MA with a methoxy functional group distal to the head group (α-MA-methoxy cis, Avanti Polar Lipids, Alabaster, Alabama, 791281P-1mg) was dried down and sonicated into 50 mM sodium citrate buffer at pH 4 containing 0.06% CHAPS. The sonicates were transferred to microfuge tubes, and 20 μl of CD1b monomer was added. GMM-loaded tetramers were incubated in a 37°C water bath for 2 hours with vortexing every 30 minutes, and MA-loaded tetramers were incubated at 37°C overnight. At the end of the incubation, the solution was neutralized to pH 7.4 with 6 μl of 1 M Tris pH 9. Finally, 25.5 μl of Streptavidin conjugated to ECD, 15.4 μl APC (Life Technologies, Carlsbad, CA, SA1017, S868), 10.2 μl BV510, or 10.2 μl BV650 (BioLegend, San Diego, CA, 405234, 405232) was added in ten aliquots every 10 minutes. The final product was filtered through a SpinX column (Sigma, St. Louis, MO, CLS8160-96EA) to remove aggregates and stored at 4°C until use.

PBS-57 (synthetic analogue of α-galactosylceramide (α-GalCer))-loaded and 5-(2-oxopropylideneamino)-6-D-ribitylaminouracil (5-OP-RU)-loaded MR1 monomers were provided by the National Institutes of Health Tetramer Core Facility (Emory University, Atlanta, GA). Tetramers were prepared as previously described (16, 37). Briefly, 10 μL of the loaded stock monomers was incubated with 28.8 μL of streptavidin conjugated to PE (Life Technologies, Carlsbad, CA, S866), or 10.2 μl BV421 (BioLegend, San Diego, CA, 405225) that were titrated in at ten aliquots of 2.88 μL or 1.02 μl every 10 min to facilitate tetramerization, respectively. The tetramers were filtered through a SpinX column (Sigma, St. Louis, MO, CLS8160-96EA) to remove aggregates and stored at 4°C until use

### *Ex Vivo* Tetramer Staining and Analysis

#### Tetramer Staining

For *ex vivo* analysis of GMM-CD1b, MA-CD1b, αGalCer-CD1d, and 5-OP-RU-MR1 tetramer positive cells, PBMC were thawed in warm thaw media (RPMI 1640 (Gibco, Waltham, MA, 11875-119) supplemented with 10% fetal bovine serum (FBS) (Hyclone, Logan, UT, SH3007003HI) and 2 μl/ml Benzonase (Millipore, Billerica, MA, 70746-3) sterile-filtered) and centrifuged at 1500 rpm for 5 minutes. The supernatant was decanted, and the cells were resuspended in RPMI 1640 (Gibco, Waltham, MA) supplemented with 10% FBS (Hyclone, Logan, UT, SH3007003HI) (R10 Media) and enumerated using the Guava easyCyte (Millipore) with guavaSoft v.2.6 software. Cells were then rested overnight at 37°C/5% CO_2_ at 2 million cells per mL in R10 media. The following day, the cells were centrifuged at 1500 rpm for 5 minutes and plated at a density of 1 million cells per well in a 96-well U-bottom plate. The PBMC in the 96-well plate were washed with FACS buffer (1x phosphate-buffered saline (PBS) (Gibco, Waltham, MA, 14190-250) supplemented with 0.2% bovine serum albumin (BSA) (Sigma, St. Louis, MO, A9418-100G)) and centrifuged at 1800 rpm for 3 minutes. Next, the cells were washed twice with PBS and stained with Green Live/Dead stain (Life Technologies, Carlsbad, CA, 50-113-7443) according to the manufacturer’s instructions. Following a 15-minute incubation at room temperature, the cells were washed twice in PBS. They were then blocked with 50% human serum (Valley Biomedical, Winchester, VA, HS1004CHI) in FACS buffer for 10 minutes at 4°C. The wells were washed twice with FACS buffer and then resuspended in 50 µl FACS buffer supplemented with 50 nM Dasatinib (Cayman Chemicals, Ann Arbor, MI, 11498) and anti-CCR7 antibody for 30min at 37°C (**Supplemental Table 2**). Cells were then washed twice with FACS buffer and then resuspended in FACS buffer supplemented with 50 nM Dasatinib (Cayman Chemicals, Ann Arbor, MI, 11498) with tetramers specified in **Supplemental Table 2**. Optimal titre for tetramer staining was determined prior to use. After this incubation period, the cells were washed twice with FACS buffer and then labelled with a cocktail containing antibodies specified in **Supplemental Table 2** in FACS buffer supplemented with 1 mM L-ascorbic acid (Sigma, St. Louis, MO, A4403-100MG) and 0.05% sodium azide (Sigma, St. Louis, MO, S2002-5g) for 30 minutes at 4°C. Antibody cocktail was centrifuged at 3,500 rpm for 5 min prior to use to remove antibody aggregates. After two final washes in FACS buffer, the cells were fixed in 1% paraformaldehyde (Electron Microscopy Sciences, Hatfield, PA, 15712-S) and acquired on a BD LSRFortessa (BD Biosciences, San Jose, CA) equipped with blue (488 nm), green (532 nm), red (628 nm), violet (405 nm), and ultraviolet (355 nm) lasers using standardized good clinical laboratory practice procedures to minimize the variability of data generated.

#### Data Analysis

The raw data were compensated and manually gated using FlowJo v9.9.6 (TreeStar Inc.). A representative gating tree is shown in **Supplemental Figure 1**. The data were then analyzed using the OpenCyto framework in the R programming environment (38). All samples met our predefined quality control criteria for good viability defined on the basis of minimum CD3 (>10,000 cells) or CD4 counts (>3,000 cells). Tetramer, CD4, CD8, CD45RA, CCR7, CD38, CD56, TRAV1-2, and HLA-DR gates for GMM-CD1b panel were defined in OpenCyto using the mindensity2 and tailgate functions (38).

### Immunosequencing and TCR Analysis

#### Immunosequencing

High-throughput sequencing of TCRs was performed using the ImmunoSEQ platform (Adaptive Biotechnologies, Inc) with the TCR-α/δ (TCRAD) assay for each sample using a multiplex PCR approach followed by Illumina high-throughput sequencing (39). Only productive templates were used in the analyses we describe here, and no minimum template count criteria was utilized as an exclusion criterion. Template frequencies for each subset of interest were aggregated in the R programming environment according to the parameters summarized in the Figure Legends.

#### Identification of Expanded Meta-Clonotypes

We identified TCR-δ clones (N=1822 at the amino acid level) expanded from (i) pre-vaccination to (ii) 3 weeks post-vaccination or 52 weeks post vaccination using the Differential Abundance tool in the Adaptive Biotechnologies ImmunoSEQ Analyzer toolkit. This tool uses a binomial method to determine expanded clonotypes, defined by an exact nucleotide sequence match (alpha = 0.01, Benjamini-Hochberg adjusted). Biochemical properties (length, hydrophobicity, bulkiness, polarity, acidity, aliphatic, basic, aromatic, charge) of the CDR3 region of expanded clonotypes were computed using the alakazam R package (version 1.0.2) (40). From the expanded clones, we computed pairwise CDR-weighted distances between all TCRDV clonotypes (defined as δ-chain amplicons identical at the amino acid level) using the Python package tcrdist3 (41) implementing the TCRdist metric (42). Briefly, for each clone we found all clones present at 3 weeks and 52 weeks within 18 TCRdist units, which is equivalent to 1-2 AA substitutions or gaps in the CDR3 junction depending on the biochemical similarity of residues substituted. We refer to these groups of similar TCRs defined by a centroid sequence and TCRdist radius as “meta-clonotypes,” which allow for quantification of similar TCRs across all the samples. We retained meta-clonotypes that were public (i.e., those formed from clones from at least two individuals). Additionally, the CDR3s of each meta-clonotype were aligned to the centroid and a position-specific motif pattern of conserved amino-acid residues was defined to further restrict meta-clonotype membership when searching for similar sequences in bulk repertoires (41). Alignment positions with five or fewer distinct amino acids were considered conserved. The motif constraint is permissive of substitutions in select positions relative to the centroid, however these substitutions are penalized by the radius constraint. Where a gap exists, that position was made optional in the motif. The motif was encoded as a regular expression, with the “.” character indicating non-conserved positions and specified degenerate amino acid indicated by the set of allowable residues in brackets (e.g., “D[ST][LV]LGDT[RG]T?DKL”). All tested meta-clonotypes are summarized in **Supplemental File 1** (see supporting information), as they may provide useful priority, and likely donor-unrestricted, features for independent validation in separate BCG study cohorts.

#### Beta Binomial Models

Using the identified meta-clonotypes (above), we computed the distance between each meta-clonotype cluster centroid and all the clones in each of the bulk TCR-δ repertoire samples. For each bulk repertoire sample, we tabulated the number of templates associated with each meta-clonotype, i.e., those that were ≤ 18 TCRdist from the centroid TCRDV-CDR3 search sequence and matching the position-specific CDR3 motif. For each bulk repertoire sample, we also computed the effective sequence depth as the number of total templates associated with TCR-δ chain amplicons. For each exact-clonotype and meta-clonotype, we estimated a maximum likelihood beta-binomial model based on clonotype-counts (W) and total sample counts (M) and the visit number. The model formula was specified as cbind(W, M - W) ~ visit using the bbdml function in R package corncob 0.1.0 (43), where visit was coded as a categorical variable with values V01 (0 weeks) or V28 (52 weeks).

### Statistical Methods

Statistical tests are described in the Figure and Table legends. Categorical variables were analyzed using a Fisher’s exact test. All statistical testing was performed using non-parametric methods which does not require a normal distribution of the data. When two continuous variables were analyzed, a Wilcoxon signed rank test was used. When more than two continuous variables were analyzed, a Kruskal-Wallis test was performed. *Post-hoc* Dunn tests were performed after Kruskal-Wallis tests to determine which group(s) were different. When multiple hypotheses were tested, p-values were adjusted using the Benjamini-Hochberg method. These tests were conducted in R (v3.8.5) or Graph Pad Prism version 6 (GraphPad, GSL Biotech, San Diego, CA). No sample size determination was performed in advance. All p-values reported in the Figures and Text are adjusted for multiple comparisons. For our two flow cytometry data sets, immunosequencing data set, and expanded clone analysis 14, 5, 12, and 9 tests were conducted and corrected for, respectively. Samples were not randomized as we examined samples from a single arm of the parent trial. Researchers were not blinded to time point during sample acquisition. Sample quality control criteria are described in the *Methods* above.

## Results

### Analysis of DURT Cell Frequencies After BCG Revaccination Using Flow Cytometry and Immunosequencing

**Figure 1:**
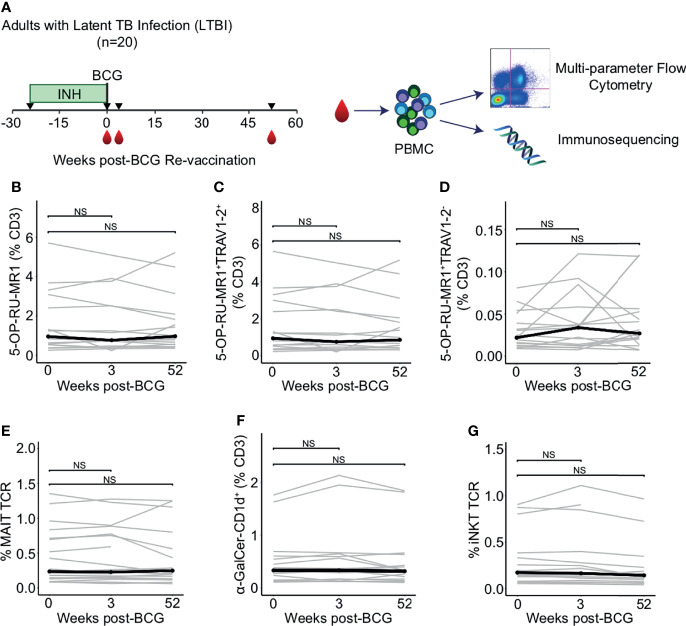
Peripheral blood frequencies of iNKT and MAIT cells are not modulated by BCG revaccination. **(A)** Archived peripheral blood mononuclear cells (PBMC) from participants in a clinical trial to test the safety and immunogenicity of BCG revaccination in South African adults (n = 20) were examined using multiparameter flow cytometry panels that include CD1 and MR1 tetramers and a γδ T cell antibody to assess changes in donor unrestricted T (DURT) cell abundance. PBMC were analyzed at the time of revaccination, 3 weeks post-revaccination, and 52 weeks post-revaccination. Genomic DNA was extracted for immunosequencing. **(B)** Percentage of 5-OP-RU-MR1 tetramer positive T cells **(C)** Percentage of 5-OP-RU-MR1 tetramer positive and TRAV1-2 positive, or **(D)** Percentage of 5-OP-RU-MR1 tetramer positive and TRAV1-2 negative cells were quantified in PBMC isolated 0 weeks, 3 weeks, and 52 weeks post-revaccination. Percentages are expressed as a percent of CD3 positive cells in each sample. Connected lines depict the percentage of 5-OP-RU-MR1 tetramer positive cells from each participant at each time point (grey). The median percentage from each time point is shown (black). **(E)** Percent of templates that contain TRAV1-2, TRAJ33, and have a CDR3 length of 12 amino acids to identify canonical MAIT TCR-α chains is expressed as a percent of total productive templates at each time point. **(F)** Percentage of α-GalCer-CD1d tetramer positive T cells expressed as a percent of CD3 positive cells in each sample. **(G)** Percent of templates that contain TRAV10, TRAJ18, and have a CDR3 length of 15 amino acids to identify canonical MAIT TCR-α chains is expressed as a percent of total templates at each time point. Statistical testing was performed using the Wilcoxon signed-rank test with Benjamini-Hochberg correction for multiple hypothesis testing and adjusted p-values are shown. NS, Not Significant.

We leveraged samples from a phase I randomized controlled trial in healthy South African adults with a positive (>= 15 mm induration) tuberculin skin test (TST), all of whom were vaccinated with BCG at birth and randomized to six months of isoniazid preventive therapy (IPT) or placebo prior to revaccination with BCG at week 0 (**Supplemental Table 1**) (30). Only samples from subjects that received IPT were analyzed here (**Figure 1A**). PBMC were collected and archived at several time points, including at 0 weeks pre-vaccination, 3 weeks post- revaccination (3 weeks), and 52 weeks post-revaccination (52 weeks). We performed comprehensive profiling of DURT cells using multiparameter flow cytometry and T cell receptor sequencing (**Supplemental Table 2**, **Table 1**).

**Table 1:** Immunosequencing Summary Statistics.

Metric	0 weeks	3 weeks	52 weeks
Number of Samples	19	19	19
Median Productive Template Count	246947	226885	241711
Range Productive Template Count	4001-323646	44613-288099	59139-346643
Mean Productive Simpson Clonality	0.0526	0.0458	0.0561

Immune repertoire profiling was performed using the TCRAD ImmunoSEQ platform (Adaptive Biotechnologies, Inc.). All metrics are summarized by time point. Productive templates were defined as in-frame sequences with no premature stop codon. Productive Simpson Clonality is calculated for a sample as the square root of Simpson’s diversity index for all productive rearrangements. Values range from 0 to 1, where 1 represents a monoclonal sample, and 0 represents a highly polyclonal sample.

### Peripheral Blood Frequencies of iNKT and MAIT Cells Are Not Modulated by BCG Revaccination

The frequency of MAIT cells, as defined by T cells staining with 5-OP-RU-MR1 tetramer and with anti-TRAV1-2, did not change between 0 weeks and 3 weeks or 52 weeks-post BCG revaccination (p = 0.523 and p = 0.720, respectively, Wilcoxon signed-rank) (**Figures 1B, C**; **Supplemental Figure 1** and **Supplemental Table 2**). We also observed that MR1-restricted T cells that do not utilize TRAV1-2 were not different at either the 3 week or 52 week time point compared to 0 weeks (p = 0.496 and p = 0.589, respectively, Wilcoxon signed-rank) (**Figure 1D**). We used immunosequencing to compare the frequency of the canonical MAIT cell TCR-α chain rearrangement, which is defined by the use of TRAV1-2 and TRAJ33 and a fixed CDR3 length of 12 amino acids. Again, we found no difference in clonotype frequencies between the 0 week and the 3 week or 52 week time point (p = 0.936 and p = 0.756, respectively, Wilcoxon signed-rank) (**Figure 1E**).

Next, we compared the frequency of iNKT cells between 0 weeks and 3 weeks or 52 weeks as defined by staining with α-GalCer-CD1d tetramer or as a TCR-α chain rearrangement of TRAV10, TRAJ18, and a CDR3 length of 15 amino acids. We were unable to detect a change in frequency of iNKT cells between 0 weeks and 3 weeks or 52 weeks by either flow cytometry (p = 0.496 and p = 0.901, respectively, Wilcoxon signed-rank), or immunosequencing (p = 0.936 and p = 0.417, respectively, Wilcoxon signed-rank) (**Figures 1F, G**). Taken together, these data reveal that innate-like T cells that do not specifically recognize mycobacterial antigens do not undergo clonal expansion after BCG re-vaccination in humans with a positive TST.

### Peripheral Blood Frequencies of Mycolipid-Specific T Cells Are Not Modulated by BCG Revaccination

**Figure 2:**
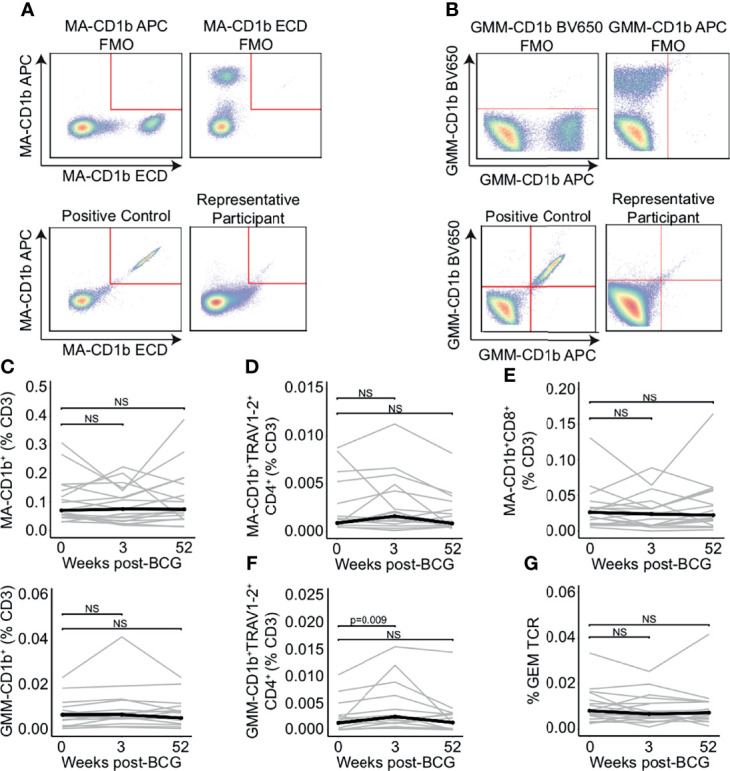
Peripheral blood frequencies of mycolipid-specific T cells are not modulated by BCG revaccination. CD1b tetramers loaded with mycolic acid (MA-CD1b) or glucose monomycolate (GMM-CD1b) were incorporated into a multi-parameter flow cytometry assay to measure changes in the frequency of mycolipid-specific T cells following BCG revaccination. **(A)** The tetramer positive gate was defined by a dual tetramer staining with electron coupled dye (ECD) and allophycocyanin (APC) and ‘Fluorescence Minus One’ (FMO) negative control (top) and a positive control using MA-specific T cell line diluted in donor PBMC (bottom left). Representative MA-CD1b tetramer staining from one participant is shown (bottom right). **(B)** The GMM-CD1b tetramer positive gate was defined by a dual tetramer staining with Brilliant Violet 650 (BV650) and allophycocyanin (APC) and ‘Fluorescence Minus One’ (FMO) negative control (top) and a positive control using GMM-specific T cell line diluted in donor PBMC (bottom left). Representative GMM-CD1b tetramer staining from one participant is shown (bottom right). **(C)** Percentage of MA-CD1b and GMM-CD1b tetramer positive T cells, **(D)** MA-CD1b tetramer positive, TRAV1-2 positive, and CD4 positive, and **(E)** MA-CD1b tetramer positive and CD8 positive T cells were quantified. Percentages are expressed as a percent of CD3 positive cells and connected lines depict the percentage of MA-CD1b tetramer positive cells from each participant at each time point (grey). The median percentage from each time point is shown (black) **(F)** Percentage of GMM-CD1b tetramer positive, TRAV1-2 positive, and CD4 positive T cells were quantified. Percentages are expressed as a percent of CD3 positive cells in each sample. **(G)** Percent of GEM templates that contain TRAV1-2, TRAJ9-1, and have a CDR3 length of 13 amino acids, expressed as a percent of total productive templates at each time point. Statistical testing was performed using the Wilcoxon signed-rank test with Benjamini-Hochberg correction for multiple hypothesis testing, and adjusted p-values are shown. NS, Not Significant.

To enumerate the frequencies of mycolipid-specific T cells, we modified a highly sensitive flow cytometry assay that we have previously developed for human immune monitoring studies (35). We used a dual-tetramer labelling strategy incorporating tetramers loaded with two mycobacterial glycolipid antigens, mycolic acid (MA) and glucose monomycolate (GMM), that were each labelled with two distinct fluorochromes. MA- and GMM-specific T cells were defined as staining with both tetramers while being unlabeled by a mock-loaded CD1b tetramer containing endogenous lipids (**Figures 2A, B**; **Supplemental Figure 1**) (44, 45). Gates defining MA- and GMM-CD1b tetramer-positive cells were set based on staining human MA- and GMM-specific T cell lines and fluorescence minus one (FMO), or no tetramer, controls, thus ensuring highly sensitive and specific detection of mycolipid-specific T cells (**Figures 2A, B**; **Supplemental Figure 2**) (35). MA- and GMM-specific T cells were computationally defined as staining with both antigen-loaded tetramers while being unlabeled by a mock-loaded CD1b tetramer containing endogenous lipids. Representative staining from one individual at week 0 is shown (**Figures 2A, B**).

We did not detect a change in the percentage of MA-CD1b and GMM-CD1b tetramer positive cells between 0 and 3 or 52 weeks post BCG-revaccination (p = 0.496 and p = 0.589, respectively, and p = 0.168 and p = 0.962, respectively, Wilcoxon signed-rank) (**Figure 2C**). Further, we did not detect a difference in the percentage of MA-specific GEM T cells, as defined by co-staining with either the MA-CD1b tetramer, TRAV1-2, and CD4, and the percentage of MA-specific CD8^+^ T cells between week 0 and 3 weeks or 52 weeks post-BCG revaccination (p = 0.496 and p = 0.502, respectively, Wilcoxon signed-rank) (**Figures 2D, E**). However, when we compared the percentage of GMM-specific GEM T cells between 0 weeks and 3 weeks post-BCG revaccination, we did detect an increase in GMM-specific GEM T cells from approximately 0.001% to 0.003% of T cells (p = 0.009, Wilcoxon signed-rank), which was not present after 52 weeks (p = 0.962, Wilcoxon signed-rank) (**Figure 2F**). This finding suggested a modest and transient expansion of mycolipid-specific T cells after BCG vaccination. To confirm this finding, we quantified the frequency of the canonical GEM TCR-α chain sequences, as defined by rearrangements containing TRAV1-2, TRAJ9, and a CDR3 length of 13 amino acids (46, 47). We did not detect a statistically significant difference in the frequency of GEM clonotypes between the week 0 sample and 3 weeks or 52 weeks post-BCG revaccination (p = 0.936 and p = 0.756, respectively, Wilcoxon signed-rank) (**Figure 2G**). These conflicting data suggest that some mycolipid-specific T cells expressing a non-canonical TCR-α sequence may be transiently expanded after BCG revaccination.

### TCR-δ Clonotypes Are Durably Expanded After BCG Revaccination

**Figure 3:**
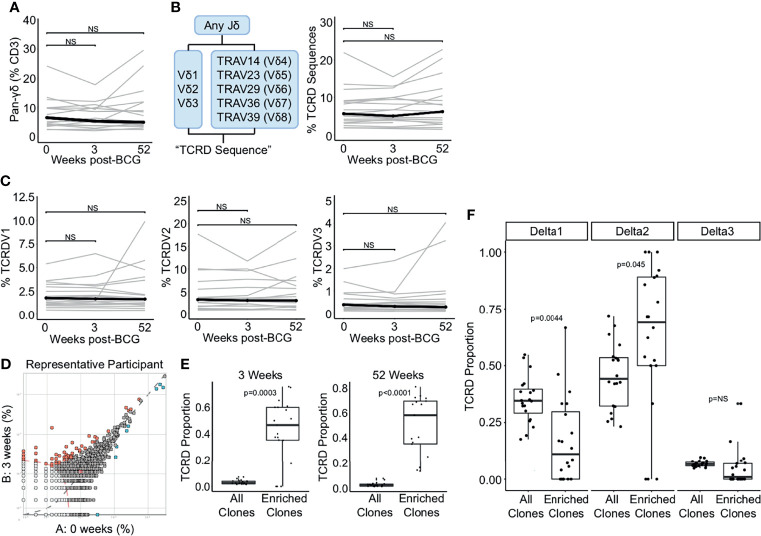
TCR-δ clonotypes are durably expanded after BCG revaccination. **(A)** A pan-γδ antibody (clone 11F2) was used to quantify changes in the frequency of γδ T cells following BCG revaccination. Percentage of γδ T cells in PBMC isolated 0 weeks, 3 weeks, and 52 weeks post-revaccination. Percentages are expressed as a percent of CD3 positive cells in each sample. Connected lines depict the percentage of γδ T cells from each participant at each time point (grey). The median percentage from each time point is shown (black). **(B)** γδ TCRs were quantified as the percent of templates that contain a TRDJ and TRDV1, TRDV2, TRDV3, TRAV14 (TRVD4), TRAV23 (TRDV5), TRAV29 (TRDV6), TRAV36 (TRDV7), or TRAV39 (TRDV8). This is expressed as a percent of total templates. **(C)** Percent of templates that contain TRDV1 (left), TRDV2 (center), or TRDV3 (right) expressed as a percent of total templates at each time point. **(D)** The differential abundance tool (ImmunoSEQ analyzer) was used to identify clones that are expanded post-BCG revaccination. Scatter plot visualizes the frequency of clones at the 0 week time point (x-axis) and 3 weeks post-BCG (y-axis) from one representative participant. Each dot represents one clone. Red dots are statistically enriched at 3 weeks, and blue dots are statistically enriched at the 0 week time point (Binomial test with Benjamini-Hochberg correction for multiple hypothesis testing, n = 130,727 clones). **(E)** Proportion of expanded clones that express TCR-δ compared to the proportion of all clones that express TCR-δ at 3 weeks (left) and 52 weeks post-BCG (right). Each dot represents one participant (Wilcoxon signed-rank test, n = 18, p = 0.0003 and p < 0.0001, respectively). **(F)** Proportion of expanded TCR-δ clones that contain TCRDV1 (left), TCRDV2, (center), or TCRDV3 (right) compared to the proportion of all TCR-δ clones at 3 weeks post BCG that contain each respective TCRDV gene. Each dot represents one participant (p = 0.004, 0.045, and 0.079, respectively, n = 18). Unless stated otherwise, statistical testing was performed using the Wilcoxon signed-rank test with Benjamini-Hochberg correction for multiple hypothesis testing, and adjusted p-values are reported. NS, Not Significant.

Finally, we examined the effect of BCG revaccination on γδ T cells. We did not detect a change in the frequency of total γδ T cells between the 0 and 3 weeks or 52 weeks post-BCG revaccination using flow cytometry (p = 0.496 and p = 0.589, respectively, Wilcoxon signed-rank) (**Figure 3A**). We used immunosequencing to quantify TCR-δ frequency by including only those sequences containing a rearrangement that uses any TCRDJ gene paired with TCRDV1, TCRDV2, TCRDV3, TRAV14 (TCRDV4), TRAV23 (TCRDV5), TRAV29 (TCRDV6), TRAV36 (TCRDV7), or TRAV39 (TCRDV8) (48) (**Figure 3B**). Using this definition, we detected no change in the frequency of total TCR-δ sequences criteria between the 0 weeks and 3 weeks or 52 weeks post-BCG revaccination timepoints (p = 0.936 and p = 0.756, respectively, Wilcoxon signed-rank) (**Figure 3B**). Further, when we examined the frequency of sub-populations that utilize TCRDV1, TCRDV2, and TCRDV3, we also found no statistically significant changes in total frequencies over time (TCRDV1: p = 0.936 and p = 0.756, respectively, TCRDV2: p = 0.936 and p = 0.756, respectively, and TCRDV3: p = 0.936 and p = 0.756, respectively, Wilcoxon signed-rank) (**Figure 3C**). These data suggest that the total frequency of γδ T cells in the blood is not affected by BCG revaccination in humans with a positive TST.

Having thus queried the frequencies of iNKT, MAIT, mycolipid-specific, and γδ T cells in a targeted manner, we next took an ‘unsupervised’ approach to identify any/all clonotypes that were expanded after BCG vaccination. We selected for TCR clonotypes (as defined by an exact match for V gene, J gene, and CDR3 sequence) that were significantly expanded post-revaccination (**Figure 3D**). In this plot from a representative individual, relatively few sequences are expanded, and only clones that are expanded at the 3 week or 52 week time point were included in the subsequent analyses (**Figure 3D**). Importantly, a mean of 56% and 50% of expanded clones across all individuals met the criteria outlined above for TCR-δ sequences, emphasizing a significant enrichment above all clones at the 3 week and 52 weeks timepoints, respectively, and highlighting the durable nature of this expansion (p = 0.0003 and p < 0.0001, respectively, Wilcoxon signed-rank) (**Figure 3E**). To extend these findings we next compared the gene usage of TCR-δ expanded clones to TCR-δ gene usage of all TCR-δ clones and detected a significant enrichment of TCRDV2, which was detected in 71% of expanded clones and only 49% of TCR-δ clones at the 3 week time point (p = 0.045, Wilcoxon signed-rank) (**Figure 3F**). This enrichment was accompanied by a reduction in TCRDV1 usage among TCR-δ expanded clones, which was reduced to 11% from 31% (p = 0.004) (**Figure 3F**). We detected no significant enrichment in TCRDV3 frequency among expanded clones (p = 0.079, Wilcoxon signed-rank) (**Figure 3F**). These data reveal that although γδ T cells as a group are not significantly perturbed by BCG revaccination, subpopulations of γδ T cells appear to be expanded in a sustained manner.

### TCR-δ Meta-Clonotypes Reveal a Donor-Unrestricted Signature of BCG Revaccination

**Figure 4:**
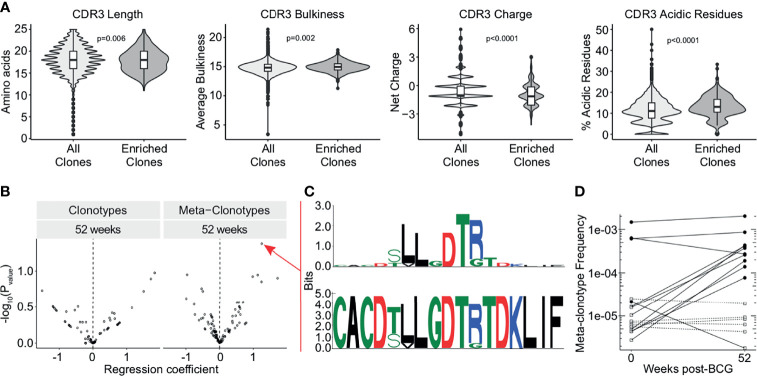
TCR-δ meta-clonotypes reveal a donor-unrestricted signature of BCG revaccination. Sequences of expanded clones were analyzed for CDR3 biochemical properties and sequence motifs. **(A)** CDR3 length (left), bulkiness (middle left), charge (middle right), and acidic residues (right) were assessed within TCR-δ expanded clones compared to CDR3 regions from all TCR-δ sequences (Wilcoxon rank-sum test with Benjamini-Hochberg correction for multiple hypothesis testing, n = 51,723 sequences). **(B)** Testing for association of TCR-δ features with post-vaccination time point in bulk repertoires of 17 BCG revaccinated individuals. Volcano plots show beta-binomial regression coefficient estimates (x-axis) and -log10 P values (y-axis). Positive coefficient estimates indicate clonotype or meta-clonotype more likely to be detected at 52 weeks post-revaccination compared to pre-revaccination. **(C)** CDR3 sequence motif of the most significantly post-vaccine associated meta-clonotype TCR feature, quantified as the sum of templates in the repertoire within 18 TCRdist units from centroid sequence and matching the motif pattern. The top sequence logo shows the information relative to amino acids found in a background set of randomly sampled TRDV2*01 and TRDJ*01 CDR3 sequences. **(D)** Meta-clonotype frequency (y-axis) in 0 weeks and 52 weeks post-revaccination samples. Empty square symbols denote non-detections plotted at 1/10th the minimum sample frequency (a function of each sample’s sequencing depth). Dashed lines indicate samples where the meta-clonotype was not detected in either the pre- or post-vaccination sample. Black circles indicate samples where the meta-clonotype was detected, and white squares indicate samples where the meta-clonotype was not detected.

As we detected this enrichment of TCRDV2 sequences among expanded clones, we next investigated whether the complementarity determining region 3 (CDR3) sequences of TCR-δ expanded clones at the 3 week time point were enriched for any biochemical properties compared to all TCR-δ clones from this time point. We compared CDR3 length, hydrophobicity, bulkiness, polarity, charge, and the proportion of aliphatic, aromatic, basic, and acidic residues (**Figure 4A** and **Supplemental Figure 3**). We found that expanded TCR-δ clones had on average a 0.27 amino acid increase in CDR3 length, a 0.16 unit increase in bulkiness, and an increase of acidic residue content by 2.0%, but had a reduced overall charge by 0.35 units (p = 0.0065, p = 0.0022, p < 0.0001, and p < 0.0001, respectively, Wilcoxon Rank Sum, n = 51,723 sequences) (**Figure 4A**). Other parameters were not significantly different (**Supplementary Figure 3**).

To examine durably expanded clones, we identified meta-clonotypes, groups of highly biochemically similar TCRs, that were expanded in more than one individual at the 52 week timepoint compared to baseline (see Methods). Each meta-clonotype was defined as a ‘neighborhood’ of biochemically similar TCRs using tcrdist3 (41). We then used these meta-clonotypes to identify and quantify TCRs in the bulk repertoires at each timepoint. We tested whether individual clonotypes or meta-clonotypes were more likely to be detected in samples 52 weeks post-vaccination relative to baseline (**Figure 4B**). After adjustment for multiple comparisons, we could not infer that any meta-clonotypes were significantly increased or decreased in abundance after BCG vaccination (q-value >0.2 or <0.2).

However, within this cohort, the meta-clonotype with the most individually significant association (p = 0.04) at 52 weeks post-revaccination was TRDV*02+CACDTLLGDTRTDKLIF (**Figure 4C**). This meta-clonotype was detected in 11 participants overall, in 4 of 17 pre-revaccination samples and 10 of 17 samples at 52 weeks post-revaccination samples. Further, this meta-clonotype increased in frequency in 9 samples and decreased in 2 samples (**Figure 4D**). This sequence contains enriched hydrophobic residues upstream of the diversity (D) region of the CDR3 that have been previously shown to be associated with Vγ9Vδ2 T cell phosphoantigen reactivity, including in the context of live mycobacterial infection (49, 50). Together, these data suggest that BCG vaccination leads to durable expansions in γδ T cells that may be specific for mycobacterial phosphoantigens.

## Discussion

In summary, we comprehensively profiled DURT cells after BCG revaccination in a cohort of South African adults and found no evidence that total frequencies as measured by flow cytometry were changed. However, analysis of the TCR repertoire clearly revealed that BCG induces clonal expansion of a subset of γδ T cells that is still detectable up to one year after vaccination. Further, we were able to identify a public meta-clonotype with shared biochemical properties that increased in frequency in nine of twenty participants following BCG revaccination. While whole cell mycobacterial vaccines are known to induce protective T cells that target mycobacterial proteins, our data reveal that BCG also induces long-term expansion of T cells targeting non-peptide antigens.

To our knowledge, this study is among the first to evaluate the effect of BCG on T cell responses to mycobacterial glycolipid antigens in humans. Previous studies have attempted to do this *via* cross-sectional study designs using historical cohorts (51). Instead, we used longitudinally collected samples from an interventional clinical trial. We also employed lipid-loaded CD1 tetramers, which we incorporated into a validated assay for immune monitoring studies (35). Despite the rigor of our approach, we did not detect any changes in the frequency of T cells targeting mycolic acid and only a transient increase in the frequency of GEM T cells that was not recapitulated by immunosequencing. One explanation for these negative findings is that the route of immunization may affect how DURT cells respond to BCG. We and others have recently demonstrated clonal expansion of MAIT cells, γδ T cells, and CD1-restricted T cells in the blood and lungs of non-human primates after intravenous administration of BCG (26, 52), and intravenous BCG induces greater expansion of Vγ9Vδ2 T cells than intradermal or aerosol vaccination (52). Another possibility is that the responses present at baseline and after treatment with isoniazid may still have been too high to observe any changes after vaccination (53). The participants we chose to study had previously been vaccinated with BCG, and had a positive TST >= 15mm, suggesting that they were infected with M.tb. High levels of pre-existing immunity prior to BCG revaccination may have impeded our ability to detect clonal expansion. A recently published study directly addresses this limitation by studying infants, who are mycobacteria naïve, yet arrived at a similar conclusion regarding γδ T cell expansion after BCG vaccination (54). However, even infants are not truly immunologically naive to antigens that modulate DURT cells. Vγ9Vδ2 T cells differentiated into effector cells within the first 10 weeks after birth, and this was likely driven by environmental antigen exposure and was not affected by BCG vaccination (55). Nevertheless, BCG vaccination at birth does lead to an expansion of total γδ T cells, supporting the possibility that non-Vγ9Vδ2 T cells are preferentially affected in this clinical setting (54).

The stability of MAIT and iNKT cell frequencies following BCG revaccination was unanticipated given the body of literature implicating MAIT and iNKT cells in the T cell response to BCG (24, 29, 33). However, this may be a product of the unique biology of DURT cells, such as high precursor frequency, memory phenotype acquisition during T cell development, and the recognition of endogenous ligands (56). Further, it is possible that the kinetics of MAIT and iNKT cell expansion are highly transient, and the timing of sample collection was not optimal to detect these changes. In addition, recently stimulated iNKT cells can exhibit hyporesponsiveness for at least one month following stimulation, which due to the chronically infected nature of these participants, may have affected their proliferative capacity (24, 57). Further, iNKT and MAIT cells have been shown to have reduced proliferative potential compared to conventional CD4 and CD8 T cells (58), which may have contributed to their lack of expansion after BCG. A recent study revealed that the protein subunit vaccine H4:IC31, induces T cells that co-express CD3 and CD56 and showed that *in vitro* restimulation of T cells by BCG elicits responses from DURT cells (59). Previous work has also demonstrated that BCG revaccination transiently expanded peripheral blood frequencies of BCG-reactive IFN-γ+ MAIT cells, indicating that the functional profiles of DURT populations may be affected, while the frequency may remain unchanged (33). The simultaneous identification of DURTs by tetramer and their functions by flow cytometry is technically challenging but could be circumvented by single-cell transcriptional profiling (56). These data reveal that BCG may modulate iNKT and MAIT cells independently of changes in total frequencies as a result of clonal expansion.

This hypothesis is supported by our results examining γδ T cells as well as a recent study examining MAIT cells after human challenge with *Salmonella paratyphi*. Following infection, the total frequency of MAIT cells did not change appreciably, but the frequency of activated MAIT cells increased as did the frequency of specific clonotypes identified by deep-sequencing the TCR-β repertoire of sorted T cells (60). As the biased but variant TCR-β chain in DURT populations can affect antigen response, it is possible that expansion of particular TCR-β clonotypes occurred following BCG revaccination that we were unable to quantify without first sorting the DURT cells (18, 61). In addition, non-canonical MR1 and CD1d-restricted T cell populations may be modulated by BCG revaccination, such as non-MAIT MR1-restricted T cells (MR1Ts) and Type II NKT cells with diverse TCRs. Our study was not designed to detect modulation of T cell populations with diverse TCR usage and ligand recognition patterns, and these populations are of interest to further evaluate upon characterization of potential ligands and TCR properties (62–64). Finally, epigenetic changes in myeloid cells following BCG vaccination (trained immunity) may be part of the mechanism by which BCG vaccination exerts its protective effect in children and adolescents (65, 66). Similar mechanisms may be at work to modify the functional potential of iNKT and MAIT cells following BCG vaccination.

We provide clear evidence that BCG modulates the γδ T cell repertoire independently of changes in total frequencies, which would be expected for conventional MHC-restricted T cells (67). TCR-δ clonotypes that were durably expanded after BCG revaccination expressed Vδ2 more frequently and were characterized by similar biochemical features (increased CDR3 lengths, acidic and bulky residue content, and a reduced charge) compared to unenriched sequences, suggesting these T cells could be targeting specific antigens present in mycobacteria. Given the small sample size and the requirement for multiplicity adjustment across all meta-clonotypes, this study was not powered to identify population-level induction of meta-clonotypes by BCG or avoid confounding by clinical and demographic factors, such as INH pretreatment, in this population of antigen-experienced individuals. However, the meta-clonotype we describe here shares conserved hydrophobic amino acids that have been previously described to be important in phosphoantigen recognition by Vγ9Vδ2 T cells, suggesting a possible antigenic target for this T cell population (48–50). In a previous study, nearly all BCG- and isopentenyl pyrophosphate-expanded Vγ9Vδ2 T cell clones sequenced expressed a hydrophobic or neutral amino acid residue in the 5’ portion of the CDR3 sequence (50). Further, a subset of Vγ9Vδ2 T cells was found to be specifically induced by BCG and shown to be more cytolytic than those derived from canonical phosphoantigen stimulation (50). The effect was shown to be mediated by methylglucose-containing lipopolysaccharides (mGLP) present in the mycobacterial cell wall (68). In addition, immunization of macaques with (E)-4-hydroxy-3-methyl-but-2-enyl pyrophosphate (HMBPP) elicits Vγ2Vδ2 T cell responses and reduces TB infection following challenge (69). The antigen specificity of the expanded T cells we describe here and their contribution to protective immunity remains to be determined.

Recent studies have revealed that BCG revaccination of adolescents or intravenous BCG vaccination of non-human primates can prevent M.tb infection (52, 70). These data raise the question of which aspects of the immune response to BCG are required for protection against M.tb. Our results are notable for showing no modulation in the total frequency of iNKT cells, MAIT cells, and CD1b-restricted T cells. These negative data are important findings in the context of immune correlates of protection but also the confounded literature describing the effect of BCG on these T cell populations in humans (32, 33). By contrast, our study revealed expansion of γδ T cells following BCG revaccination, thus highlighting this DURT subset as an important target for further study in the context of BCG as well as other novel vaccine strategies.

## Data Availability Statement

Immunosequencing data (indexed by participant identification number) are available for download from Adaptive Biotechnologies at the following: DOI, 10.21417/CAJ2020FI; URL, https://clients.adaptivebiotech.com/pub/james-2022-fi. Analysis code can be accessed at https://github.com/seshadrilab/DURT_BCG_Revaccination.

## Ethics Statement

The studies involving human participants were reviewed and approved by the Medicines Control Council (MCC, now the South African Health Products Regulatory Agency) of South Africa, Human Research Ethics Committee (HREC) of the University of Cape Town (Ref. 387/2008), University Hospitals Cleveland Medical Center, and University of Washington Institutional Review Board. The trial was registered on ClinicalTrials.gov (NCT01119521). The Human Research Ethics Committee (HREC) of the University of Cape Town also approved protocols for blood collection from participants (Ref. 177/2011). Researchers adhered to the World Medical Association’s Declaration of Helsinki and Good Clinical Practice (GCP) guidelines during the treatment of all participants. The patients/participants provided their written informed consent to participate in this study.

## Author Contributions

CS and TJS conceived of the study. EDL and KKQY performed all the flow cytometry experiments and isolated genomic DNA for immunosequencing. CAJ, MTS, and KKQY, analyzed and verified the data and generated the figures. JLJ and WAH conducted the adult BCG-revaccination trial and TJS facilitated access to archived PBMC and clinical data. AF-G and KM-B analyzed the immunosequencing data to identify expanded meta-clonotypes. CAJ and CS wrote and edited manuscript with contributions from all authors. All authors contributed to the article and approved the submitted version.

## Funding

This work was supported by the U.S. National Institutes of Health (R01 AI125189-04 to CS), the Doris Duke Charitable Foundation (Grant No. 2016103 to CS), and the Bill and Melinda Gates Foundation (Grant No. OPP1109001 to WH). The adult BCG revaccination study was funded by the U.S. National Institutes of Health (NO1-AI70022 to WH).

## Conflict of Interest

The authors declare that the research was conducted in the absence of any commercial or financial relationships that could be construed as a potential conflict of interest.

The handling editor RC declared a shared affiliation with the authors CS, MTS, CAJ, EDL, and KKQY at the time of review.

## Publisher’s Note

All claims expressed in this article are solely those of the authors and do not necessarily represent those of their affiliated organizations, or those of the publisher, the editors and the reviewers. Any product that may be evaluated in this article, or claim that may be made by its manufacturer, is not guaranteed or endorsed by the publisher.

## Supplementary Material

Supplementary Figure 1 Gating strategies for multiparameter flow cytometry panels used to identify DURT cell populations. **(A)** The gating strategy proceeded from Live and CD3^+^ events to CD14^-^ and CD19^-^ events to single cell events to keeper gates using CCR7 and 5-OP-RU-MR1 tetramer to remove staining artefact to lymphocytes by size gating. After this point, gates were drawn for γδ T cells, TRAV1-2, CD4 and CD8, and CD45RA and CCR7 gates were drawn independently for CD4 and CD8 T cells. Then, gates were drawn for 5-OP-RU-MR1 and α-GalCer-CD1d were defined by ‘Fluorescence Minus One’ (FMO) negative controls (data not shown) and tetramer staining from PBMC of a representative participant is shown (right). MA-CD1b tetramer-positive events as defined in the main text. From here, the gating set was imported into OpenCyto and cell count information was extracted for Boolean subsets of interest. **(B)** The gating strategy proceeded from Live and CD3^+^ events to CD14^-^ and CD19^-^ events to single cell events to keeper gates using CCR7 and CD38 to remove staining artefact to lymphocytes by size gating. After this point, gates were drawn for TRAV1-2 on T cells, and gates for HLA-DR and CD38 were drawn on CD3-negative cells and gates for CD45RA and CCR7 were drawn on CD3-positive cells (data not shown). CD56 gate was drawn on Live cells. Gates were drawn for GMM-CD1b tetramers as defined in the main text. The gating set was imported into OpenCyto and cell count information was extracted for subsets of interest.

Supplementary Figure 2 Validation of MA-CD1b-specific T cell line. **(A)** MA-specific T cell line was isolated from a QFT-positive donor using MA-loaded CD1b tetramer. **(B)** MA-specific T cell line was validated following *in vitro* expansion to ensure staining with MA-CD1b tetramer but not with unloaded CD1b tetramer. **(C)** MA-specific T cell line was tested for antigen-specificity using IFN-γ ELISPOT. T cells were incubated with K562 cells that were stably transfected with CD1b (K562-CD1b) or mock transfected with an empty vector (K562-EV) to control for non-specific activation by K562 cells. T cells and K562 cells were co-cultured in the presence or absence of 1 µg/mL MA to control for non-specific activation by MA and to determine sufficiency of MA and CD1b in activating this T cell clone. T cell activation was measured by quantifying the number of IFN-γ spots present in each co-culture condition. Data are representative of two independent experiments. **(D)** 80% of T cells in MA-specific T cell line co-stain with MA-CD1b tetramer and anti-TRAV1-2 antibody. 98% of T cells in MA-specific T cell line stain with anti-TRBV5-5 antibody from IOTest BetaMark assay (Beckman Coulter)

Supplementary Figure 3 Sequences of expanded clones were analyzed for CDR3 biochemical properties and sequence motifs. CDR3 hydrophobicity (top left), basic residues (top right), aliphatic residues (middle left), aromatic residues (middle right), and polarity (bottom) were assessed within TCR-δ expanded clones compared to CDR3 regions from TCR-δ sequences within (Wilcoxon rank-sum test with Benjamini-Hochberg correction for multiple hypothesis testing, n = 51,723 sequences).
