# Supplementary figures and images for: Durable Expansion of TCR-δ Meta-Clonotypes After BCG Revaccination in Humans
Source: Front Immunol. 2022 Mar 30;13:834757. doi: 10.3389/fimmu.2022.834757 (PMC9005636; doi:10.3389/fimmu.2022.834757)

**a.**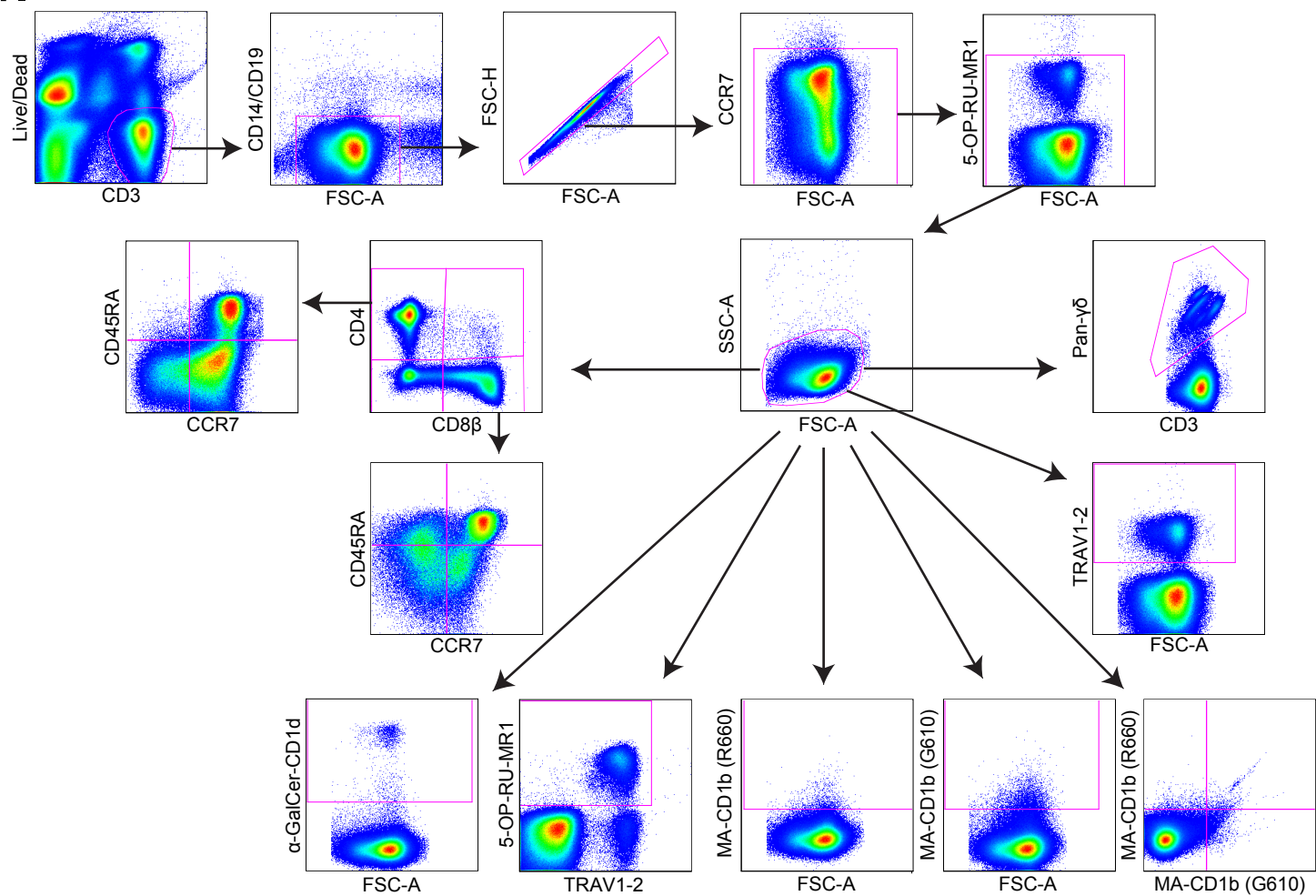**b.**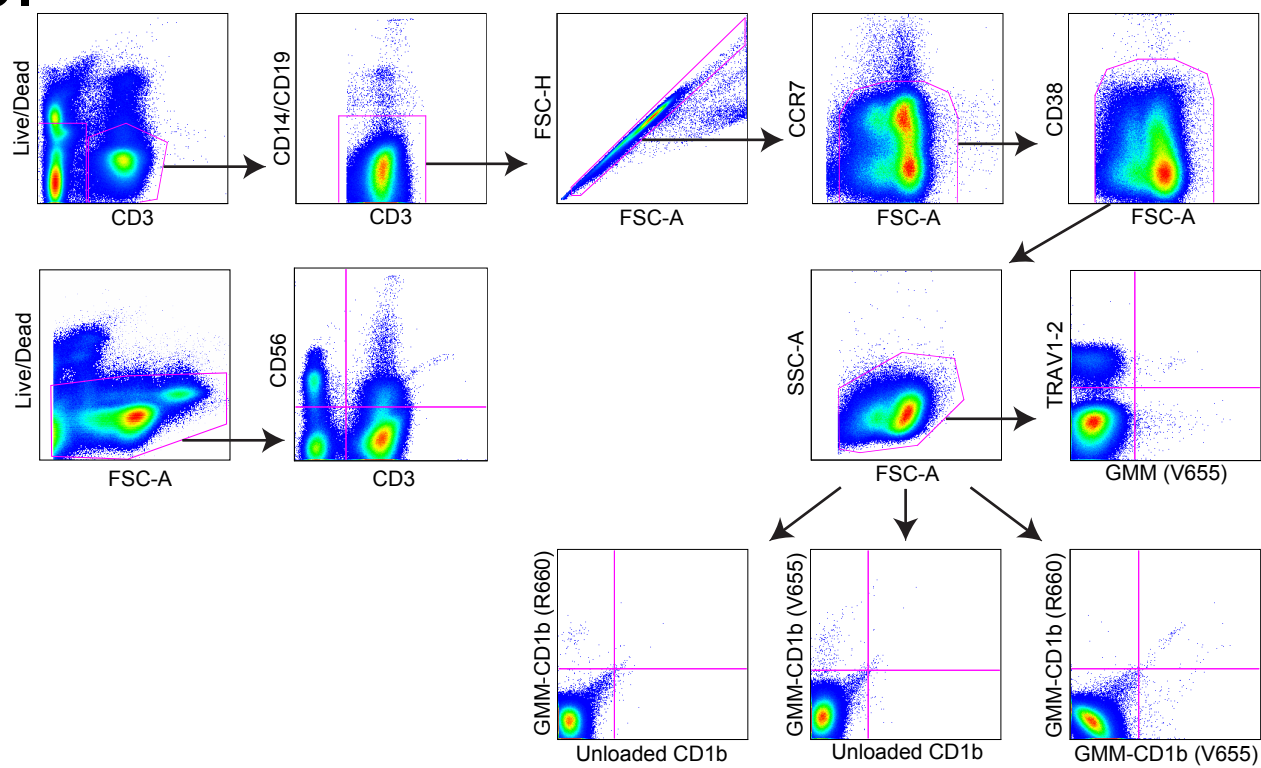

Supplement: Supplementary Figure 1 — Gating strategies for multiparameter flow cytometry panels used to identify DURT cell populations. (A) The gating strategy proceeded from Live and CD3+ events to CD14- and CD19- events to single cell events to keeper gates using CCR7 and 5-OP-RU-MR1 tetramer to remove staining artefact to lymphocytes by size gating. After this point, gates were drawn for γδ T cells, TRAV1-2, CD4 and CD8, and CD45RA and CCR7 gates were drawn independently for CD4 and CD8 T cells. Then, gates were drawn for 5-OP-RU-MR1 and α-GalCer-CD1d were defined by ‘Fluorescence Minus One’ (FMO) negative controls (data not shown) and tetramer staining from PBMC of a representative participant is shown (right). MA-CD1b tetramer-positive events as defined in the main text. From here, the gating set was imported into OpenCyto and cell count information was extracted for Boolean subsets of interest. (B) The gating strategy proceeded from Live and CD3+ events to CD14- and CD19- events to single cell events to keeper gates using CCR7 and CD38 to remove staining artefact to lymphocytes by size gating. After this point, gates were drawn for TRAV1-2 on T cells, and gates for HLA-DR and CD38 were drawn on CD3-negative cells and gates for CD45RA and CCR7 were drawn on CD3-positive cells (data not shown). CD56 gate was drawn on Live cells. Gates were drawn for GMM-CD1b tetramers as defined in the main text. The gating set was imported into OpenCyto and cell count information was extracted for subsets of interest. [file Image_1.pdf]

a.

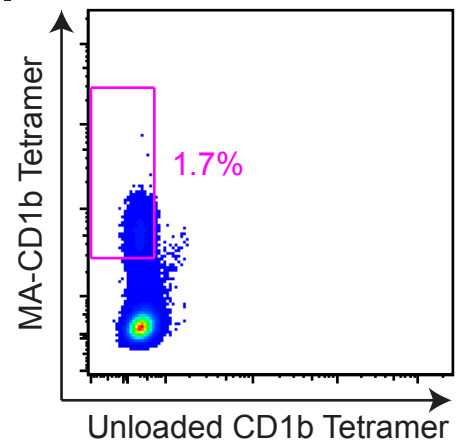

b.

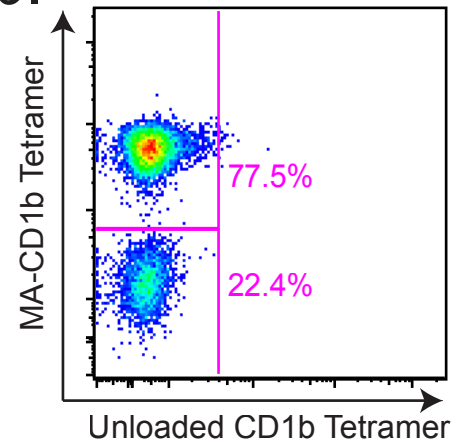

c.

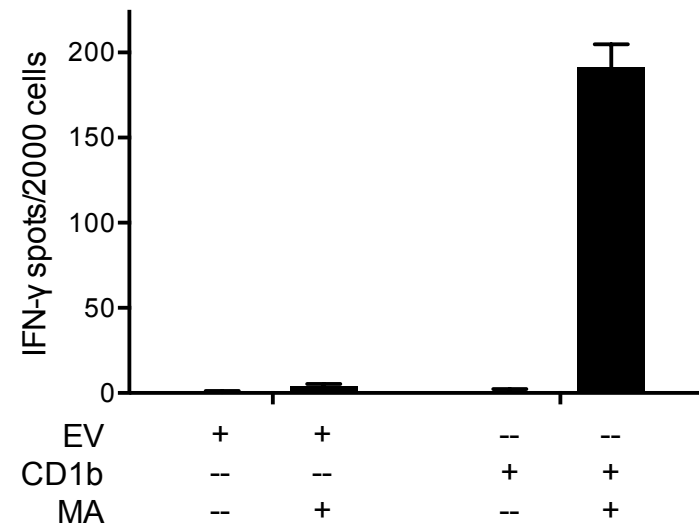

d.

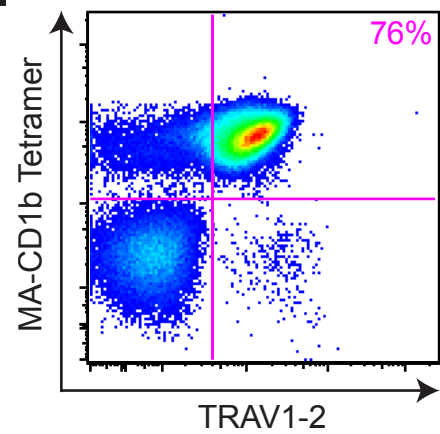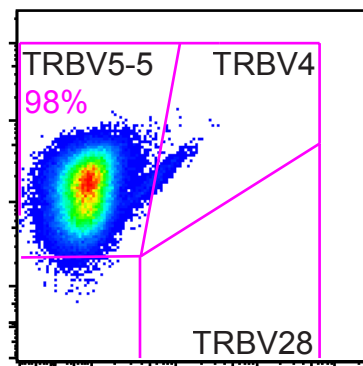

Supplement: Supplementary Figure 2 — Validation of MA-CD1b-specific T cell line. (A) MA-specific T cell line was isolated from a QFT-positive donor using MA-loaded CD1b tetramer. (B) MA-specific T cell line was validated following in vitro expansion to ensure staining with MA-CD1b tetramer but not with unloaded CD1b tetramer. (C) MA-specific T cell line was tested for antigen-specificity using IFN-γ ELISPOT. T cells were incubated with K562 cells that were stably transfected with CD1b (K562-CD1b) or mock transfected with an empty vector (K562-EV) to control for non-specific activation by K562 cells. T cells and K562 cells were co-cultured in the presence or absence of 1 µg/mL MA to control for non-specific activation by MA and to determine sufficiency of MA and CD1b in activating this T cell clone. T cell activation was measured by quantifying the number of IFN-γ spots present in each co-culture condition. Data are representative of two independent experiments. (D) 80% of T cells in MA-specific T cell line co-stain with MA-CD1b tetramer and anti-TRAV1-2 antibody. 98% of T cells in MA-specific T cell line stain with anti-TRBV5-5 antibody from IOTest BetaMark assay (Beckman Coulter) [file Image_2.pdf]
