# Supplementary material for: Durable Expansion of TCR-δ Meta-Clonotypes After BCG Revaccination in Humans
Source: Front Immunol. 2022 Mar 30;13:834757. doi: 10.3389/fimmu.2022.834757 (PMC9005636; doi:10.3389/fimmu.2022.834757)
Supplement: Supplementary Figure 3 — Sequences of expanded clones were analyzed for CDR3 biochemical properties and sequence motifs. CDR3 hydrophobicity (top left), basic residues (top right), aliphatic residues (middle left), aromatic residues (middle right), and polarity (bottom) were assessed within TCR-δ expanded clones compared to CDR3 regions from TCR-δ sequences within (Wilcoxon rank-sum test with Benjamini-Hochberg correction for multiple hypothesis testing, n = 51,723 sequences). [file Image_3.pdf]

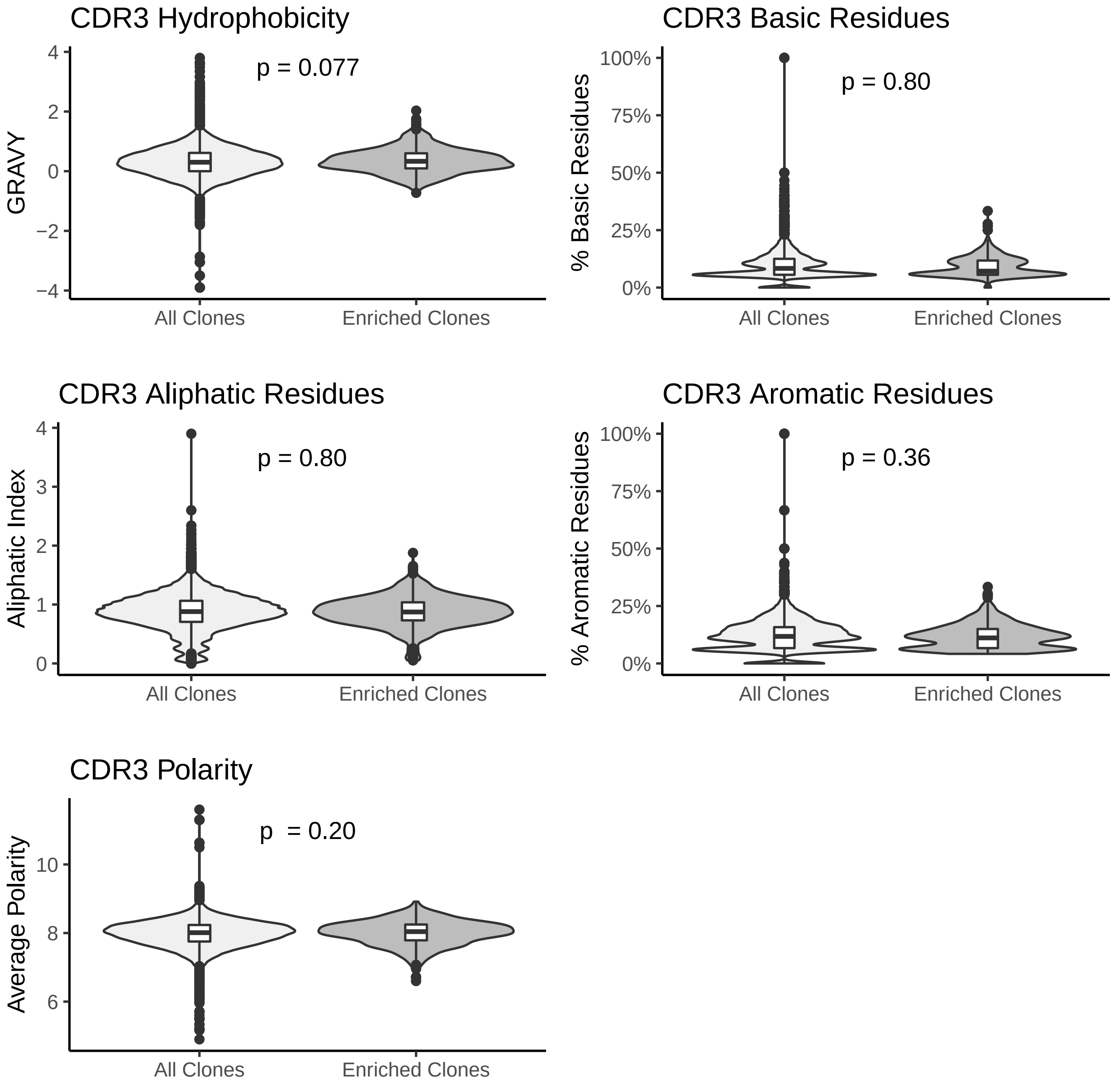

CDR3 Hydrophobicity
GRAVY
p = 0.077
4
2
0
−2
−4
All Clones
Enriched Clones
CDR3 Basic Residues
% Basic Residues
p = 0.80
100%
75%
50%
25%
0%
All Clones
Enriched Clones
CDR3 Aliphatic Residues
Aliphatic Index
p = 0.80
4
3
2
1
0
All Clones
Enriched Clones
CDR3 Aromatic Residues
% Aromatic Residues
p = 0.36
100%
75%
50%
25%
0%
All Clones
Enriched Clones
CDR3 Polarity
Average Polarity
p = 0.20
10
8
6
All Clones
Enriched Clones
