# Supplementary material for: Durable Expansion of TCR-δ Meta-Clonotypes After BCG Revaccination in Humans
Source: Front Immunol. 2022 Mar 30;13:834757. doi: 10.3389/fimmu.2022.834757 (PMC9005636; doi:10.3389/fimmu.2022.834757)
Supplement: Supplementary file 4 [file Table_1.pdf]

Supplemental Table 1. Participant Demographic Information

Participant ID	Age (Years)	Sex	TST (mm)	BMI (kg/m^2)	Flow Cytometry			ImmunoSEQ		
					0 weeks	3 weeks	52 weeks	0 weeks	3 weeks	52 weeks
0023	37	F	24	26.9	X	X	X	X	X	X
0033	24	M	25	22.2	X	X	X	X	X	X
0048	25	F	23	37.6	X	X	X	X	X	X
0052	24	M	17	21.9	X	X	X	X	X	X
0071	20	M	23	22.1	X	X	X	X	X	X
0086	19	F	15	18.4	X	X	X	X	X	
0093	27	F	18	27.0	X	X	X	X	X	X
0096	26	F	21	34.4	X	X	X	X	X	X
0109	23	M	19	18.0	X	X	X	X	X	X
0122	38	F	29	36.5	X	X	X	X	X	X
0131	39	F	16	40.9	X	X	X	X	X	X
0155	24	F	15	27.2	X	X	X	X	X	X
0159	19	F	19	23.9	X	X	X	X	X	X
0164	19	F	18	24.3	X	X	X			X
0173	21	F	19	17.6	X	X	X	X	X	X
0189	25	M	22	21.8	X	X	X	X	X	X
0224	22	M	23	18.1	X	X	X	X	X	X
0228	20	F	27	26.3	X	X	X	X	X	X
0238	20	F	26	29.9	X	X	X	X	X	X
0241	22	M	16	18.9	X	X	X	X	X	X

Supplemental Table 1. Summary of samples used for *ex vivo* analysis of DURT cells following BCG revaccination. Samples used in this study are listed by participant identifier (ID) and were derived from a clinical trial designed to determine safety and immunogenicity of BCG revaccination in South African adults with latent TB (Hatherill et al., 2014). Age reflects the age at last birthday at the time the sample was collected. Sex is self-reported. TST induration at study enrolment is reported in millimetres (mm). Body mass index **(**BMI) was calculated from height (cm) and weight (kg) obtained from physical examination during office visit. The Flow Cytometry and ImmunoSEQ columns summarize which assays were performed on each sample. A blank cell indicates that no sample was included from this time point.
