# Supplementary material for: Durable Expansion of TCR-δ Meta-Clonotypes After BCG Revaccination in Humans
Source: Front Immunol. 2022 Mar 30;13:834757. doi: 10.3389/fimmu.2022.834757 (PMC9005636; doi:10.3389/fimmu.2022.834757)
Supplement: Supplementary file 5 [file Table_2.pdf]

Supplemental Table 2. Flow Cytometry panels.

Flow Panel 1

Fluorophore	Detector	Specificity	Antibody Clone	Manufacturer (Catalogue #)	Purpose
BB700	B710	CD8β	2ST8.5H7	BD Biosciences (745761)	Lineage
FITC	B515	Live/Dead	-	Life Technologies (L23101)	Viability
PE-Cy7	G780	CD45RA	HI100	BioLegend (304125)	Memory
ECD	G610	CD3	UCHT1	Beckman Coulter (IM2705U)	Lineage
PE	G575	Mock-CD1b	-	Life Technologies (SA1017)	Exclusion
APC-Alx750	R780	CD4	13B8.2	Beckman Coulter (A94685)	Lineage
Alx700	R710	CD56	HCD56	BioLegend (318316)	Lineage
APC	R660	GMM-CD1b	-	Life Technologies (S868)	Antigen-specificity
BV780	V780	CD14 & CD19	M5E2 & SJ25C1	BioLegend (301840, 363028)	Exclusion
BV711	V710	CCR7	150503	BD Biosciences (566602)	Memory
BV650	V655	GMM-CD1b	-	BioLegend (405232)	Antigen-specificity
BV605	V610	HLA-DR	G46-6	BD Biosciences (562845)	Activation
BV510	V510	TRAV1-2	3C10	BioLegend (351717)	TCR Identification
BV421	V450	CD38	HIT2	BD Biosciences (562444)	Activation

Flow Panel 2

Fluorophore	Detector	Specificity	Antibody Clone	Manufacturer (Catalogue #)	Purpose
BB700	B710	CD8β	2ST8.5H7	BD Biosciences (745761)	Lineage
FITC	B515	Live/Dead	-	Life Technologies (L23101)	Viability
PE-Vio770	G780	Pan-$\gamma\delta$	11F2	Miltenyi Biotec (130-113-505)	TCR identification
ECD	G610	MA-CD1b	-	Life Technologies (SA1017)	Antigen-specificity
PE	G575	PBS57-CD1d	-	Life Technologies (SA1017)	Antigen-specificity
APC-Alx750	R780	CD4	13B8.2	Beckman Coulter (A94685)	Lineage
APC	R660	MA-CD1b	-	Life Technologies (S868)	Antigen-specificity
BV780	V780	CD14 & CD19	M5E2 & SJ25C1	BioLegend (301840, 363028)	Exclusion
BV711	V710	CCR7	150503	BD Biosciences (566602)	Memory
BV605	V610	TRAV1-2	3C10	BioLegend (351720)	TCR Identification
BV510	V510	Mock-CD1b	-	BioLegend (405234)	Antigen-specificity
BV421	V450	5-OP-RU-MR1	-	BioLegend (405225)	Antigen-specificity
BUV737	U730	CD45RA	HI100	BD Biosciences (564442)	Memory
BUV395	U395	CD3	UCHT1	BD Biosciences (563546)	Lineage
